# Moral distress and burnout in caring for older adults during medical school training

**DOI:** 10.1186/s12909-020-1980-5

**Published:** 2020-03-23

**Authors:** Subha Perni, Lauren R. Pollack, Wendy C. Gonzalez, Elizabeth Dzeng, Matthew R. Baldwin

**Affiliations:** 1 Department of Radiation Oncology, Harvard Medical School, Boston, MA USA; 2 Department of Medicine, University of Washington, Seattle, WA USA; 3 Department of Medicine, Columbia University Vagelos College of Physicians and Surgeons, New York, NY USA; 4 Department of Medicine, University of California, San Francisco, CA USA

**Keywords:** Education medical, Geriatrics, Moral distress, Burnout, Surveys and questionnaires

## Abstract

**Background:**

Moral distress is a reason for burnout in healthcare professionals, but the clinical settings in which moral distress is most often experienced by medical students, and whether moral distress is associated with burnout and career choices in medical students is unknown. We assessed moral distress in medical students while caring for older patients, and examined associations with burnout and interest in geriatrics.

**Methods:**

A cross-sectional survey study of second-, third-, and fourth-year medical students at an American medical school. The survey described 12 potentially morally distressing clinical scenarios involving older adult patients. Students reported if they encountered each scenario, and whether they experienced moral distress, graded on a 1–10 scale. We conducted a principal axis factor analysis to assess the dimensionality of the survey scenarios. A composite moral distress score was calculated as the sum of moral distress scores across all 12 scenarios. Burnout was assessed using the Maslach Abbreviated Burnout Inventory, and interest in geriatrics was rated on a 7-point Likert scale.

**Results:**

Two-hundred and nine students responded (47%), of whom 90% (188/209) reported moral distress in response to ≥1 scenario with a median (IQR) score of 6 (4–7). Factor analysis suggested a unidimensional factor structure of the 12 survey questions that reliably measured individual distress (Cronbach alpha = 0.78). Those in the highest tertile of composite moral distress scores were more likely to be burnt out (51%) than those in the middle tertile of scores (34%), or lowest tertile of scores (31%) (*p* = 0.02). There was a trend towards greater interest in geriatrics among those in the higher tertiles of composite moral distress scores (16% lowest tertile, 20% middle tertile, 25% highest tertile, *p-for-tend =* 0.21). Respondents suggested that moral distress might be mitigated with didactic sessions in inpatient geriatric care, and debriefing sessions with peers and faculty on the inpatient clerkships on medicine, neurology, and surgery, where students most often reported experiencing moral distress.

**Conclusions:**

Moral distress is highly prevalent among medical students while caring for older patients, and associated with burnout. Incorporating geriatrics education and debriefing sessions into inpatient clerkships could alleviate medical student moral distress and burnout.

## Background

Moral distress is a negative emotional state that results when a person feels inhibited from addressing a situation felt to be ethically problematic due to external constraints, including hierarchical or institutional constraints [1]. Prior studies show that American medical students frequently experience moral distress on clinical rotations [2, 3], and most often when caring for older adult patients [4]. Medical students may be susceptible to experiencing moral distress while caring for older adults because it involves complex medical decision making and end-of-life care [5], and because medical students spend a significant amount of time with patients, but lack clinical-decision making power compared to resident or attending physicians [6]. Furthermore, caring for older adult patients may be a frequent source of moral distress for medical students because older adults, defined as age ≥ 65 years, represent a large proportion of the patient population, regardless of clinical rotation or medical specialty [7].

Among nurses, resident physicians, and attending physicians, moral distress is associated with decreased professional quality-of-life and burnout [8–20], and burnout is associated with poor mental health and lower quality of patient care [21]. The association between moral distress and burnout has not yet been studied in medical students. However, medical students have reported thoughts of dropping out of medical school or choosing a non-clinical specialty due to moral distress [22].

Collectively, these prior studies suggest that moral distress in caring for older adults may be one of many reasons why the number of medical graduates choosing a subspecialty career in geriatrics is inadequate and declining [23]. By 2025 there will be a shortage of nearly 27,000 geriatricians in the United States [24]. Physicians who care for adults, regardless of specialty, will increasingly have to manage complex care decisions for their older adult patients, and therefore may be at greater risk for experiencing moral distress and its negative consequences. Accordingly, to better prepare medical students to care for older adults with complex medical needs, we first need to identify the clinical settings in which medical students most often experience moral distress with treating older patients, determine whether moral distress is associated with burnout and career choices, and explore coping strategies for medical student moral distress.

Therefore, we conducted a cross-sectional survey study of medical students to identify clinical settings where medical students experienced moral distress while caring for older adult patients, and sought to determine whether mortal distress is associated with burnout and decreased interest in geriatric career paths. We also investigated strategies that medical students used to cope with moral distress, and elicited suggestions for interventions to treat medical student moral distress.

## Methods

### Study design, participants, and setting

We conducted a cross-sectional, web-based survey study of medical students at the Columbia University Vagelos College of Physicians and Surgeons. Students received didactic training in bioethics through a humanism in medicine course, but there is no dedicated course in geriatrics or palliative care. Clerkships occur at Columbia University Medical Center and affiliate community hospitals. The survey was distributed between February and June of 2016 by e-mail invitation. Eligible participants included all second-, third-, and fourth-year medical students. Second year students began major clinical year rotations in January and were surveyed between March and June; thus they had 3–5 months of experience caring for patients prior to completing the survey. The survey was administered using Qualtrics Software (Qualtrics, Provo, Utah), and responses were anonymized. Participation was voluntary, and incentivized by reward of a six-dollar gift card upon completion. All students first provided informed consent electronically prior to taking the suvery. The Columbia University Medical Center Institutional Review Board approved this study (protocol AAAQ4215).

### Measurements

#### Moral distress

We measured moral distress through responses to a series of 12 challenging and common clinical scenarios involving the clinical care of older adults, who were defined as patients age ≥ 65 years (see Table 1). Scenarios 1–4 and 8–10 were taken from the validated Moral Distress Scale for nurses [25]. Five additional scenarios were added based on the experiences of medical student investigators/authors (LRP and SP) and their discussions with their peers, with the goal of capturing a wider spectrum of scenarios in which medical students might experience moral distress.

**Table 1:** The 12 clinical scenarios experienced by medical students during the major clinical year that may invoke moral distress

Scenario Number	Scenario Prompt^a^
1	Following the family’s wishes to continue life support even though I felt it was not in the best interest of the elderly patient.
2	Administration of life-extending therapy to an elderly patient even though I felt it was futile or harmful.
3	Administration of tests or treatments that I felt would be unlikely to benefit an elderly patient (e.g. daily labs, cancer screening).
4	Performance of painful procedures on elderly patients by trainees in order to increase skill level.
5	Felt that an elderly patient’s capacity to make medical decisions was incorrectly assessed.
6	Use of physical or pharmacologic restraints for an elderly patient that I felt to be unwarranted.
7	Avoidance of communication with an elderly patient because communication was made more difficult by disability (e.g. cognitive impairment, difficulty hearing, difficulty seeing, speech impairment).
8	Lack of concern for the bodily privacy of an older patient.
9	Felt that the wishes of an elderly person regarding end-of-life care had not been asked in a timely or appropriate manner.
10	Felt that an elderly patient’s prognosis was not adequately communicated.
11	Felt that a hospitalized elderly patient did not have the financial resources or social support to adequately care for him or herself after discharge.
12	Witnessed ageist remarks or attitudes from the health care team about an elderly patient.

^a^Scenarios 1,2,3,4,8,9, and 10 were adapted from the Moral Distress Scale (25)

We defined moral distress for respondents as “recognizing the situation to be ethically problematic and feeling inhibited from doing anything about it” [1]. Survey questions and response options were the same for each of the 12 scenarios (see Supplement Table 1). Participants who responded that they had moral distress from experiencing a given scenario were asked to rate the severity of moral distress on an integer scale from 1 to 10, to identify the clinical settings and rotations during which the morally distressing experience most often occurred, and to describe their reason for feeling inhibited from intervening.

Medical students were asked about the utility of the following programmatic interventions: “Expanded or improved geriatrics education”; “Expanded or improved bioethics education”; “An outpatient clinical clerkship site focused on the care of older adult patients”; “Integrating discussions into an existing course on humanism in medicine that coincides with clinical rotations during the major clinical year”; and “Educating attending and resident physicians about addressing medical student moral distress.” They were also asked open-endedly about coping strategies.

#### Burnout

We measured burnout using the validated two-item Maslach Burnout Inventory criteria [26]. Respondents reported the frequency with which they felt “burned out from my work” (emotional exhaustion domain) or felt “more callous toward people since I started medical school” (depersonalization domain). Those reporting a frequency of “every week” in response to either item were categorized as experiencing burnout.

#### Interest in geriatric career paths

We measured interest in caring for older patients by response to the question: “How likely are you to have a career focus in aging or the care of older adults?” Responses were graded on a 7-point Likert scale ranging from 1 (Very Unlikely) to 7 (Very Likely), with 4 as “Undecided”. Those who reported that they were at least “somewhat likely” to have a career focus in aging or the care of older adults, were categorized as having interest in a geriatrics career path.

#### Covariables

Respondents were asked their age, gender, race/ethnicity. We asked about factors we believed could plausibly influence how one perceives moral distress in treating older adults: the degree to which religion was important in their daily lives; level of experience caring for older adults; whether they had close relationships with older adults; and whether they had ever been present for the hospitalization of an older relative. We asked respondents to complete the 14-item University of California Los Angeles (UCLA) Geriatrics Attitude Scale questionnaire, a validated measure of attitudes towards older adults [27]. Each item was a statement to which the student responded on a Likert scale that ranged from 1 (strongly disagree) to 5 (strongly agree), with 3 representing a neutral response. There were 9 positively-worded and 5 negatively-worded statements, with the final score calculated by reversing scores to negatively worded statements and averaging across all 14 items. Scores ranged from 1 to 5, with 5 representing the most positive attitude towards older adults.

### Statistical analyses

We conducted a principle axis factor analysis to determine if there were different latent factors that each contributed to moral distress. We assessed the dimensionality of the scale based on an evaluation of factor proportion of variance explained, eigenvalues, and factor loadings [28]. After confirming the unidimensional structure of the 12-item survey, we calculated composite moral distress scores by imputing a score of 0 for any scenarios the respondents reported not encountering, and then summing moral distress scores across all scenarios (possible range 0–120). For example, if a student reported a moral distress severity of 3 in response to scenario 1, 2 in response to scenario 2, and 5 in response to scenario 10, but did not encounter any of the other scenarios, the composite moral distress severity score would be 2 + 3 + 5 = 10. To assess the reliability of the composite score as a measure of individual moral distress, we calculated a Cronbach’s alpha in which each scenario was a product of its frequency and severity. We compared categorical variables using Chi square tests and compared continuous variables by composite moral distress score tertiles using the Anova or Kruskal-Wallis test. Outcomes were categorized as (1) burnout or no burnout based on the Maslach Burnout Inventory, and (2) interest or no interest in geriatric career paths. We used the Cochran-Armitage test for trend to test associations between the proportions of participants reporting burnout or interest across increasing tertiles of composite moral distress scores. We conducted post-hoc analyses stratified by medical school year. A *p*-value < 0.05 was considered significant. We used recursive partitioning to test for thresholds of composite moral distress severity scores associated with burnout. Lastly, authors LRP and SP analyzed free text responses regarding coping strategies by using a grounded theory thematic analysis approach, wherein they individually developed codebooks of thematic content and compared for discrepancies in identified themes until consensus was reached [29].

## Results

### Response rate and medical student characteristics

A total of 516 medical students were invited to participate, including 199 fourth-year, 172 third-year, and 143 second-year students. There was a response rate of 54% among fourth-year, 41% among third-year, and 47% among second-year medical students, for an overall response rate of 47% (245 participants). We excluded 36 respondents who started but did not complete the survey, leaving 209 respondents for analyses.

Most respondents (71%) had completed all major clinical year rotations at the time of survey completion. The median (IQR) age was 26 (25–28) years, 121 (59%) were female, and 122 (60%) were non-Hispanic white. Most (65%) reported some experience with caring for older adults in a clinical setting, and 39% reported having a very close relationship with older adult relatives or non-relatives. The median (IQR) UCLA Geriatrics Attitude Scale Score was 3.7 (3.4–4.0), reflecting neutral to positive attitudes towards older patients (Table 2).

**Table 2:** Medical student characteristics by tertile of composite moral distress score

Characteristics	*n* with data	All	Lowest Tertile^a^ of Composite Moral Distress (0–17)	Middle Tertile^a^ of Composite Moral Distress (18–35)	Highest Tertile^a^ of Composite Moral Distress (36–95)	*p-*value
Year in Medical School	199					< 0.01
Mid-Major Clinical Year(n, %)		57 (29%)	38 (62%)	18 (26%)	1 (1%)	
Post-Major Clinical Year (n, %)		142 (71%)	23 (38%)	51 (74%)	68 (99%)	
Age (median, IQR)	197	26 (25–28)	26 (24–27)	26 (25–27)	27 (25–28)	0.08
Female gender (n, %)	205	121 (59%)	33 (49%)	40 (58%)	48 (71%)	0.03
Race/Ethnicity (n, %)	202					0.90
Non-Hispanic White		122 (60%)	38 (56%)	40 (60%)	44 (66%)	
Non-Hispanic Black		12 (6%)	4 (6%)	4 (6%)	4 (6%)	
Hispanic		17 (8%)	7 (10%)	6 (9%)	4 (6%)	
East Asian		26 (13%)	10 (15%)	9 (13%)	7 (11%)	
Indian Subcontinent		10 (5%)	2 (3%)	3 (4%)	5 (7%)	
Other		15 (7%)	7 (10%)	5 (7%)	3 (5%)	
Religion is moderately or very important in daily life (n, %)	205	50 (24%)	16 (24%)	19 (28%)	15 (22%)	0.74
Experience caring for older adults in a clinical setting	209					< 0.01
Little or none (n, %)		51 (24%)	26 (37%)	17 (24%)	8 (12%)	
Some (n, %)		135 (65%)	40 (57%)	46 (66%)	49 (71%)	
A lot (n, %)		23 (11%)	4 (6%)	7 (10%)	12 (17%)	
Present for hospitalization of elderly relative (n, %)	209	124 (59%)	35 (50%)	45 (64%)	44 (64%)	0.15
Very close relationship with elderly relatives or non-relatives (n, %)	198	78 (39%)	25 (38%)	26 (39%)	27 (41%)	0.94
UCLA Geriatrics Attitude Scale Score (median, IQR) (range 1–5, 5 most positive)	208	3.7 (3.4–4.0)	3.6 (3.1–3.9)	3.7 (3.4–4.0)	3.8 (3.4–4.1)	0.05

^a^Tertile 1 ranges from 0 to 11 (*n* = 70), tertile 2 ranges from 18 to 35 (*n* = 70), tertile 3 ranges from 36 to 95, (*n* = 69)

### Moral distress

Respondents encountered a median (IQR) of 5 (3–8) of the 12 potentially morally distressing scenarios. The percentage of respondents who encountered each potentially morally distressing scenario ranged from 15% for a scenario involving the use of physical or pharmacological restraints to 76% for a scenario involving following the family’s wishes to continue life support despite feeling that it was not in the best interest of the older adult patient. Among students who reported encountering each potentially morally distressing scenario, between 86% and 100% reported feeling moral distress, depending on the specific scenario (Table 3). Overall, 90% of medical students (188/209) reported having experienced moral distress in response to at least one scenario in caring for an older adult patient with a median (IQR) severity score of 6 (4–7).

**Table 3:** Scenario characteristics reported by medical student respondents (*n* = 209)

Scenarios	1	2	3	4	5	6	7	8	9	10	11	12
Encountered the scenario, (n, %)	131 (63%)	114 (55%)	128 (61%)	79 (38%)	70 (33%)	29 (14%)	110 (53%)	54 (26%)	100 (48%)	103 (49%)	122 (58%)	58 (28%)
Experienced moral distress in response to scenario^a^, (n, %)	125 (95%)	106 (93%)	106 (83%)	68 (86%)	67 (96%)	29 (100%)	99 (90%)	51 (94%)	97 (97%)	96 (93%)	118 (97%)	50 (86%)
Score among all, median (IQR)	5 (3–6)	5 (3–6)	3 (2–5)	5 (4–7)	6 (5–7)	6 (4–7)	5 (3–6)	5 (4–7)	5 (4–7)	6 (5–7)	6 (5–8)	6 (3–6)
Score among distressed, median (IQR)	5 (3–6)	5 (4–6)	4 (2–5)	5 (4–7)	6 (5–7)	6 (4–7)	5 (3–6)	5 (4–7)	6 (4–7)	6 (5–7)	6 (5–8)	6 (4–7)
Rotation experienced on which scenario most commonly experienced^b^	Med (90%)	Med (91%)	Med (93%)	Med (84%)	Surg (67%)	Med (76%)	Med (78%)	Med (83%)	Med (96%)	Med (87%)	Med (56%)	Med (81%)
	Neuro (37%)	Neuro (39%)	Neuro (39%)	Surg (43%)	Med (56%)	Neuro (45%)	Neuro (59%)	Surg (67%)	Neuro (44%)	Surg (61%)	Neuro (39%)	Surg (59%)
	Surg (26%)	Surg (39%)	Surg (32%)	Neuro (30%)	Neuro (39%)	Surg (24%)	Surg (50%)	Neuro (52%)	Surg (44%)	Neuro (49%)	Surg (33%)	Neuro (48%)
								Gyn (26%)				
Setting in which scenario most commonly experienced^b^	ICU (76%)	Wards (68%)	Wards (89%)	Wards (92%)	Wards (99%)	Wards (83%)	Wards (97%)	Wards (96%)	Wards (95%)	Wards (94%)	Wards (100%)	Wards (98%)
	Wards (56%)	ICU (66%)	ICU (38%)	ICU (29%)	ICU (19%)	ICU (31%)	ICU (34%)	ICU (46%)	ICU (44%)	ICU (47%)	ICU (24%)	ICU (34%)
	Out-patient (2%)	Out-patient (4%)	Out-patient (19%)	Out-patient (10%)	Out-patient (11%)		Out-patient (19%)	Out-patient (15%)	Out-patient (25%)	Out-patient (27%)		Out-patient (31%)

^a^Denominator is number who encountered the scenario

^b^Percentages do not add to 100% because the choices were not mutually exclusive

Principal axis factor analysis suggested a unidimensional factor structure of the 12-item moral distress survey. Ninety percent of the proportion of the variability of the survey was explained by one factor. Only a one factor solution had an eigenvalue > 1 (3.5), with additional factor solutions having eigenvalues < 0.7. Factor loadings of 10 of the 12 scenarios were all greater than 0.4 for a single factor solution. With a two factor solution, only one scenario had a loading of > 0.4 (0.42) on the second factor (Supplement Table 2). The median (IQR) composite moral distress score based on all 12 scenarios was 26 (11–42). The composite moral distress score had good reliability (Cronbach’s alpha = 0.78).

Greater composite moral distress scores were associated with completion of clinical year rotations (*p* <  0.01), female gender (*p* = 0.03), and more clinical experience caring for older adults (*p* <  0.01) (Table 2). Morally distressing scenarios were most frequently encountered on medicine (77%), neurology (42%), and surgery rotations (39%), and almost always in the inpatient setting (99%). The frequency at which the 12 scenarios were encountered, the severity of moral distress for each scenario, and the settings in which the scenarios were most commonly experienced are summarized in Table 3.

Reasons for feeling inhibited from taking action varied by scenario, with the most common being playing a subordinate role on the team (79%), feeling as if it were futile to act (36%), and feeling as if concerns were due to incomplete knowledge and judgment (35%) (Supplement Table 3).

### Burnout

**Fig. 1:**
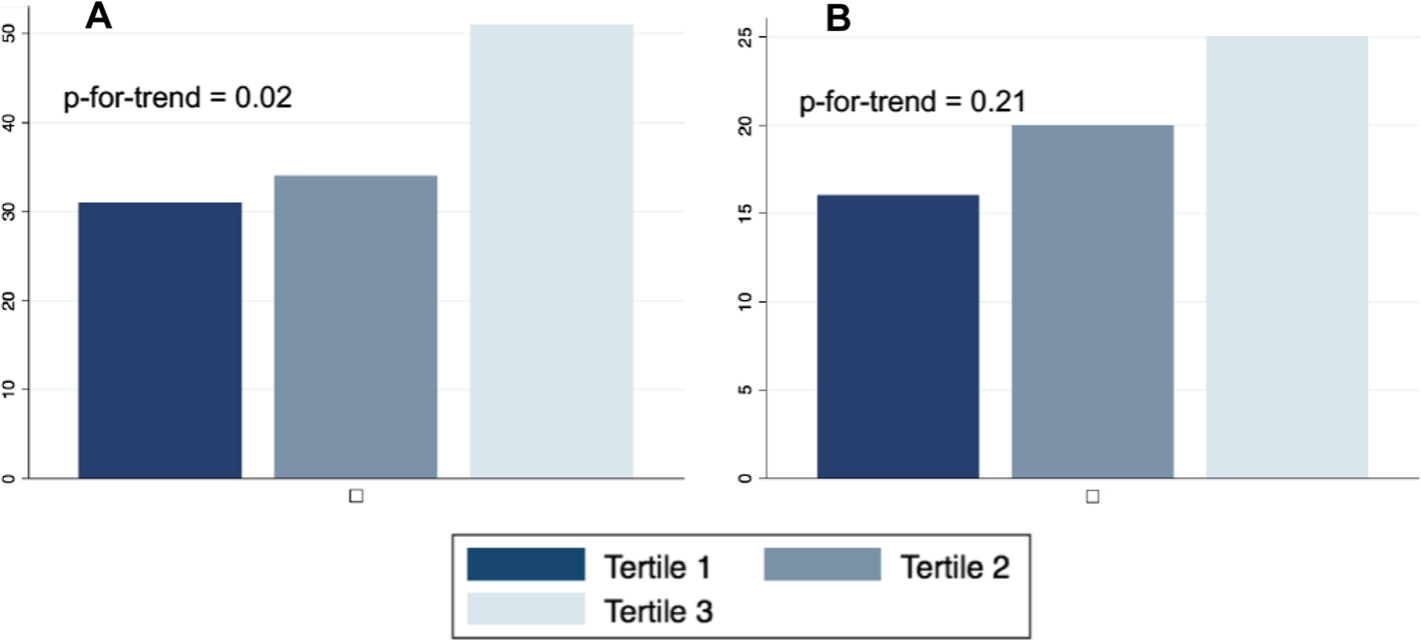
**a** Percent (number) of respondents reporting burnout by composite moral distress score tertile. **b** Percent (number) of respondents reporting interest in geriatric career paths by composite moral distress score tertile. Tertile 1 scores 0–17 (*n* = 70), tertile 2 score (18–35) (*n* = 70), and tertile 3 scores 36–95 (*n* = 69). Burnout is defined using the abbreviated Maslach burnout inventory definition [26]

A total of 81 medical students (39%) met the Maslach scale criteria for burnout. Those in the highest tertile of composite moral distress scores were more likely to be burnt out (51%) than those in the middle tertile of scores (34%) or lowest tertile of scores (31%) (*p-for-trend* = 0.02) (Fig. 1a). Compared with third and fourth year students, second year students reported lower median (IQR) composite moral distress scores (8 [3–19] versus 30 [18–43] and 29 [18–46]) and a lower prevalence of burnout (28% versus 48 and 40%). While there was less moral distress and burnout among second year students who had overall less clinical experience, the association between moral distress score and burnout did not appear to vary by medical school year (p-for-interaction = 0.62, see Supplement Table 4).

Recursive partitioning showed that those with composite moral distress scores ≥35 were 55% more likely to be burnt out than those with lower scores (RR 1.55; 95% CI 1.12–2.17), and those exposed to ≥4 scenarios were 60% more likely to be burnt out compared to those exposed to < 4 scenarios (RR 1.60; 95% CI 1.01–2.52) (Supplement Table 5).

### Interest in geriatrics

A total of 40 medical students (21%) expressed interest in a career focused on aging or the care of older adults. Respondents with higher composite moral distress scores had higher scores on the UCLA Geriatrics Attitude Scale (*p* = 0.05) (Table 2). Those in the highest tertile of composite moral distress scores more often expressed interest in geriatric career paths (25%) than those in the middle tertile of moral distress scores (20%), or lowest tertile of scores (16%), but the trend did not meet statistical significance (*p-for-trend* = 0.21) (Fig. 1b).

### Coping strategies

A total of 125 respondents (60%) wrote in free text responses identifying 165 discrete moral distress coping strategies. Coping strategies were classified thematically as: debriefing with an external network (24%), professional network (42%), or with un-specified parties (4%); private self-reflection (8%); patient alliance (14%); self-education (10%); self-care/mindfulness (10%); avoidance/inaction/rationalization (14%); and action (6%). Representative quotations organized by theme are presented in Supplement Figure 1.

The interventions identified as being useful to alleviate moral distress centered around geriatrics education and debriefing with peers and more senior physicians. Approximately half (51%) of respondents felt that improved or expanded geriatrics education would be useful, 38% felt that improved bioethics education would be useful, 32% felt that debriefing discussions during a humanism in medicine course that coincides with clinical rotations would be useful, 31% felt that educating attending and resident physicians about student moral distress would be useful, and 30% felt that the creation of an outpatient clerkship focused on the care of older adult patients would be useful.

## Discussion

In this single-center cross-sectional study of American medical students we found that 90% experienced moral distress while caring for older adult patients, and that nearly 40% experienced burnout. We found that more severe moral distress while caring for older patients was associated with burnout, as well as a trend towards an association with increased interest in geriatrics. Students attempted to cope with moral distress through debriefing, reflection, and self-education. They experienced high levels of moral distress during inpatient ward rotations, particularly in medicine, neurology, and surgery. Integration of focused didactics about inpatient geriatrics and bioethics as well as facilitated debriefing sessions regarding care of older adults into these rotations might prevent burnout and increase students’ confidence and satisfaction in caring for older adult patients.

We found that women tended to experience greater moral distress, which is consistent with prior studies [3, 30]. Further studies are needed to characterize how women experience moral distress differently than men. Other characteristics associated with greater moral distress included more positive attitudes towards older adults and more experience caring for older adults in the clinical setting. Students who have more positive attitudes towards older adults may experience greater moral distress when older patients are potentially harmed because of greater levels of sympathy or empathy. Contrary to our hypothesis, we observed a trend towards an association between experiencing higher levels of moral distress and an increased interest in geriatric career paths. One possible explanation is that moral distress actually motivated these students’ interest in geriatrics.

Students with a composite moral distress score greater than 35 or exposure to more than 4 moral distress-provoking scenarios were about 50% more likely to experience burnout. In our post-hoc stratified analyses, we also found that the association between moral distress and burnout did not vary by medical school year. Accordingly, moral distress may not abate as students obtain more clinical experience over time, but may persist or even become more severe. Our findings may reflect what is known as the “crescendo effect”, where clinicians develop a chronic “moral residue” of lingering feelings of moral distress, culminating in either persistent or progressively higher levels of moral distress upon encountering subsequent difficult clinical scenarios [31].

Our discovery of an association between medical student moral distress severity and burnout suggests that interventions targeting moral distress could have the additional benefit of reducing burnout. We found that students developed their own coping mechanisms through debriefing within their professional networks but also thought that resident and attending support was imperative in reducing moral distress. Our findings suggest that interventions targeting moral distress in medical students should be implemented at both the individual and system-wide levels, which echoes calls for concurrent individual and system-wide interventions to address burnout in palliative care providers [32, 33].

Interventions to treat moral distress in medical students might focus on facilitating the development of relationships within professional networks, expanding opportunities for guided self-reflection, and debriefing with the clinical team. Formal debriefing sessions could focus on addressing students’ feelings of subordination within the hospital hierarchy and how that might engender feelings of ethical disempowerment [34]. Students also supported expanded geriatrics and bioethics education, which might include a specific focus on discussing scenarios identified by this study as occurring frequently or provoking high levels of distress.

As part of the Moral Distress Education Project [35], investigators interviewed experts in moral distress and published best practice strategies for medical student moral distress reduction. These best-practice strategies include using a clinical ethics consultation service [36], preventative ethics or multidisciplinary team rounding, moral distress debriefings with skilled facilitators, and *Schwartz Rounds*™, a panel-based grand rounds of difficult end-of-life cases [37]. Additionally, faculty clinicians should create a compassionate clinical environment that mitigates medical student moral distress [33, 38–40]. A 2018 American College of Physicians (ACP) position paper on ethics and professionalism in medical training recommends “empowering learners to raise concerns about ethics, professionalism, and care delivery,” and for faculty to sustain a culture supportive of “encouraging discussion of ethical concerns and making values in everyday decision explicit” [41]. Our finding that about one third of medical students thought educating faculty about moral distress and having debriefing sessions were important provides evidence to support these recent ACP recommendations. Given these recent ACP recommendations and our study results, we recommend training sessions for clinical faculty to create more nurturing and less hierarchical learning environments, and to capitalize on opportunities for reflection after medical students experience potentially morally distressing clinical care.

Our moral distress survey has content and face validity insofar as students reported experiencing nearly half of the 12 clinical scenarios with 90% experiencing at least some moral distress. Furthermore, factor analysis revealed a unidimensional factor structure to the survey, and the composite moral distress score calculated from the survey had good reliability. But, our study has several limitations. We sampled American medical students who trained at a medical school in New York City. Our survey needs to be externally validated, especially with medical students from different geographic regions and other countries. Similar to a landmark medical student survey study on moral distress that had a 60% response rate [3], only about half of eligible students responded to our survey. Therefore, we cannot exclude the possibility of non-response bias. In the unlikely hypothetical scenario that every non-responder experienced no moral distress, 45% of medical students in this study would still experience moral distress with caring for older adults.

## Conclusion

Medical students experience moral distress in caring for older adults, and higher levels of moral distress are associated with burnout. Students’ responses suggest that a focused thread of didactic sessions in inpatient geriatric care and debriefing sessions with peers and faculty that are integrated into inpatient clinical clerkships on medicine, neurology, and surgery might mitigate the effects of moral distress and subsequent burnout.

## Supplementary information

**Additional file 1 Supplement Table 1**. Survey questions and response options related to moral distress in response to scenarios 1–12. **Supplement Table 2**. Principal axis factor analysis of the 12 clinical scenarios in the moral distress survey. **Supplement Table 3**. Reasons for inhibition by scenario. **Supplement Table 4**. Analyses stratified by medical school year. **Supplement Table 5**. Recursive partitioning analysis to identify the cutoffs in composite moral distress scores and number of potentially morally distressing clinical scenarios experienced during the major clinical year associated with burnout and interest in geriatrics. **Supplement Figure 1.** Moral distress coping strategies identified using grounded theory from 125 respondents who offered strategies.

## Data Availability

The datasets used and/or analyzed during the current study are available from the corresponding author on reasonable request.

## Abbreviations

ACP: American college of physicians
IQR: Interquartile range
UCLA: University of California Los Angeles
