# Supplementary material for: Moral distress and burnout in caring for older adults during medical school training
Source: BMC Med Educ. 2020 Mar 23;20:84. doi: 10.1186/s12909-020-1980-5 (PMC7092500; doi:10.1186/s12909-020-1980-5)
Supplement: Supplementary file 1 — Additional file 1 Supplement Table 1. Survey questions and response options related to moral distress in response to scenarios 1–12. Supplement Table 2. Principal axis factor analysis of the 12 clinical scenarios in the moral distress survey. Supplement Table 3. Reasons for inhibition by scenario. Supplement Table 4. Analyses stratified by medical school year. Supplement Table 5. Recursive partitioning analysis to identify the cutoffs in composite moral distress scores and number of potentially morally distressing clinical scenarios experienced during the major clinical year associated with burnout and interest in geriatrics. Supplement Figure 1. Moral distress coping strategies identified using grounded theory from 125 respondents who offered strategies. [file 12909_2020_1980_MOESM1_ESM.docx]

**SUPPLEMENT**

**Supplement Table 1**. Survey questions and response options related to moral distress in response to scenarios 1-12

**1. Have you encountered the above scenario in a clinical setting?**

[ ] Yes [ ] No

*[If respondent chooses [x] “Yes” to question 1, survey continues to question 2. If responded chooses [x] “No” to question 1, survey continues to next scenario prompt.]*

**2. Approximately how many times have you experienced or witnessed this scenario?**

[ ] 1 - 5 times [ ] 6-10 times [ ] > 10 times

**3. In which care settings have you most commonly experienced this scenario?**

[ ] Outpatient [ ] Inpatient Wards [ ] Inpatient ICU

**4. On which rotation(s) have you most commonly experienced this scenario?**

[ ] Medicine [ ] Surgery [ ] Psychiatry [ ] Neurology [ ] Gynecology [ ] Primary Care

**5. Did witnessing or experiencing this scenario cause you moral distress?**

[ ] Yes – always [ ] Yes – sometimes [ ] No – never

*[If respondent chooses [x] “Yes - always” or [x] “Yes - sometimes” to question 5, survey continues to question 6. If responded chooses [x] “No” to question 5, survey continues to next scenario prompt.]*

**6. How much moral distress did experiencing or witnessing this scenario cause you? Please choose the number that corresponds to the severity of moral distress, with 1 representing minimal distress and 10 representing severe distress.**

[ ] 1 [ ] 2 [ ] 3 [ ] 4 [ ] 5 [ ] 6 [ ] 7 [ ] 8 [ ] 9 [ ] 10

**7. Did you feel inhibited from taking action or changing behavior in response to this scenario?**

[ ] Yes [ ] No

**8. Reasons for feeling inhibited from taking action or changing behavior (check all that apply, do not check any if you did not feel inhibited).**

[ ] Because I felt taking action would be futile

[ ] Because I played a subordinate role on the team.

[ ] Because I felt my concerns or questions were due to incomplete knowledge or judgment.

[ ] Because I was constrained by time.

[ ] Because I did not want to be disrespectful to the patient’s family.

[ ] Because taking action might have negatively affected my evaluation.

[ ] Because I wanted to preserve my relationship with an attending or resident.

| **Supplement Table 2**. Principal axis factor analysis of the twelve clinical scenarios in the moral distress survey | | | | | | |
| --- | --- | --- | --- | --- | --- | --- |
| **Factor Correlations** | | | | | | |
| **Factor** | **Proportion of Variance Explained** | **Eigenvalue** |  |  |  |  |
| Factor 1 | 0.91 | 3.5 |  |  |  |  |
| Factor 2 | 0.18 | 0.68 |  |  |  |  |
| Factor 3 | 0.09 | 0.3 |  |  |  |  |
| Factor 4 | 0.07 | 0.24 |  |  |  |  |
| Factor 5 | 0.02 | 0.09 |  |  |  |  |
| Factor 6 | 0.01 | 0.03 |  |  |  |  |
| **Factor Loadings** | | | | | | |
| **Variable** | **Factor 1** | **Factor 2** | **Factor 3** | **Factor 4** | **Factor 5** | **Factor 6** |
| Scenario 1 | 0.71 | -0.22 | 0.1 | -0.07 | -0.005 | -0.01 |
| Scenario 2 | 0.56 | -0.36 | 0.03 | 0.005 | 0.11 | -0.0001 |
| Scenario 3 | 0.64 | -0.19 | -0.06 | 0.2 | 0.07 | 0.06 |
| Scenario 4 | 0.44 | 0.18 | -0.17 | 0.26 | 0.03 | 0.005 |
| Scenario 5 | 0.55 | 0.16 | 0.31 | -0.1 | -0.005 | 0.01 |
| Scenario 6 | 0.21 | 0.19 | 0.33 | 0.11 | 0.0001 | 0.04 |
| Scenario 7 | 0.58 | 0.24 | -0.17 | -0.11 | -0.08 | 0.09 |
| Scenario 8 | 0.48 | 0.27 | 0.02 | 0.12 | 0.01 | -0.09 |
| Scenario 9 | 0.6 | -0.26 | -0.08 | -0.18 | -0.06 | -0.02 |
| Scenario 10 | 0.66 | 0.04 | -0.07 | -0.06 | 0.07 | -0.05 |
| Scenario 11 | 0.53 | 0.03 | -0.02 | 0.07 | -0.22 | -0.03 |
| Scenario 12 | 0.27 | 0.42 | -0.1 | -0.19 | 0.12 | -0.0009 |

| **Supplement Table 3**. Reasons for inhibition by scenario | | | | | | | |
| --- | --- | --- | --- | --- | --- | --- | --- |
| Scenario | Reasons for Inhibition | | | | | | |
|  | Because I felt taking action would be futile. | Because I played a subordinate role on the team. | Because I was constrained by time. | Because I did not want to be disrespectful to the patient’s family. | Because taking action might have negatively affected my evaluation. n (%) | Because I wanted to preserve my relationship with an attending and/or resident. | Because I felt that my concerns or questions were due to incomplete knowledge and judgment. |
|  | n (%) | n (%) | n (%) | n (%) |  | n (%) | n (%) |
|  |  |  |  |  |  |  |  |
| 1 (n=116) | 32 (28) | 102 (88) | 9 (8) | 65 (56) | 19 (16) | 22 (19) | 41 (35) |
| 2 (n=96) | 38 (40) | 84 (88) | 5 (5) | 45 (47) | 18 (19) | 28 (29) | 41 (43) |
| 3 (n=88) | 27 (31) | 77 (88) | 2 (2) | 22 (25) | 29 (33) | 36 (41) | 39 (44) |
| 4 (n=57) | 17 (30) | 41 (72) | 5 (9) | 5 (9) | 24 (42) | 32 (56) | 7 (12) |
| 5 (n=52) | 20 (3) | 40 (77) | 8 (15) | 12 (23) | 15 (29) | 25 (48) | 24 (46) |
| 6 (n=25) | 8 (32) | 18 (72) | 1 (4) | 1 (4) | 4 (16) | 7 (28) | 12 (48) |
| 7 (n=79) | 28 (35) | 60 (76) | 30 (38) | 5 (6) | 24 (30) | 28 (35) | 20 (25) |
| 8 (n=35) | 9 (26) | 30 (86) | 6 (17) | 4 (11) | 10 (29) | 16 (46) | 2 (6) |
| 9 (n=69) | 23 (33) | 59 (86) | 11 (16) | 17 (25) | 15 (22) | 20 (29) | 22 (32) |
| 10 (n=90) | 19 (21) | 75 (83) | 12 (13) | 23 (26) | 22 (24) | 45 (50) | 55 (61) |
| 11 (n=88) | 58 (66) | 47 (53) | 21 (24) | 10 (11) | 14 (16) | 15 (17) | 24 (28) |
| 12 (n=46) | 19 (41) | 35 (76) | 3 (7) | 0 (0) | 19 (41) | 33 (71) | 3 (7) |

| **Supplement Table 4**. Analyses stratified by medical school year. | | | | | | | | |
| --- | --- | --- | --- | --- | --- | --- | --- | --- |
| Medical school year | Composite moral distress score, median (IQR) | Moral distress score tertile 1 (n, %) | Moral distress score tertile 2 (n, %) | Moral distress score tertile 3 (n,%) | n (%) with Burnout | OR (95% CI) | p-for-association | p-for-interaction* |
| 2 | 8 (3-19) | 39 (66%) | 19 (32%) | 1 (2%) | 18 (28%) | 1.34 (0.46-3.91) | 0.6 | 0.635 |
| 3 | 30 (18-43) | 13 (22%) | 23 (38%) | 24 (40%) | 30 (48%) | 1.27 (0.65-2.49) | 0.49 |  |
| 4 | 29 (18-46) | 19 (21%) | 31 (34%) | 40 (44%) | 37 (40%) | 1.30 (0.75-2.25) | 0.83 |  |
| *interaction between medical school year and tertile of moral distress score. | | | | | | | | |

| **Supplement Table 5**. Recursive partitioning analysis to identify the cutoffs in composite moral distress scores and number of potentially morally distressing clinical scenarios experienced during the major clinical year associated with burnout and interest in geriatrics | | | | | |
| --- | --- | --- | --- | --- | --- |
|  |  | RPA Cutoff | n ≥ Cutoff | Relative Risk (95% CI) | *p* value |
| Burnout* | Sum of MD severity | 35 | 71 (34%) | 1.55 (1.12-2.17) | 0.0114* |
|  | Number of scenarios | 4 | 150 (72%) | 1.60 (1.01-2.52) | 0.0278* |
| *Burnout was defined using the abbreviated Maslach burnout inventory. | | | | | |

| **Supplement Figure 1.** Moral distress coping strategies identified using grounded theory from 125 respondents who offered strategies. | |
| --- | --- |
| **Them** | **Quotations** |
| **Debriefing (70%)**  **External Network (24%)** | Talking with people I trust about the different influences at play to try to understand why I felt constrained to act out what I thought was morally correct. |
| **Professional Network (42%)** | Discussion with peers; it was helpful to know that others were going through similar dilemmas though general consensus seemed to be that such moral dilemma was difficult to resolve due to our roles as medical students |
|  | I talk about it at length with my friends and vow to be different when I'm a resident / attending. Many of the problems feel like system-level problems, and it is hard to know how to overcome those types of problems. It usually helps me to talk about my distress with my friends or residents. |
|  | Frequent debriefing with my classmates. I think having conversations about it has helped the most. I can leave a good conversation more confident in how I want to provide care for my patients and how I can provide better care than what is sometimes modeled. |
| **Private Self-Reflection (8%)** | [T]hinking about how I would go about things differently down the road |
|  | Thought about it on my own, read articles |
|  | Tried to look at things from different angles to put myself in the shoes of all parties involved rather than casting judgment from my novice point of view. |
| **Patient Alliance (14%)** | Discussions with patients, asking them how they feel they've been treated or if there was anything I can do. |
|  | Spent more time trying to learn about older patients and to listen to their stories. This did help to relieve my distress, because it helps to humanize the patients and to remind me how unique each older patient is. |
|  | Spending more time talking to them about their lives and things other than their medical problems, getting to know them. |
| **Self-Education (10%)** | reading and learning more about how to effectively care for elderly patients |
|  | [I] just asked questions in order to understand why certain decisions were made. |
|  | I also took a bioethics elective to help better understand end of life decision making, DNR/living will status, etc. I'm also doing research into the role that goals-of-care discussions play on decision-making and outcomes |
| **Self-Care/Mindfulness (10%)** | Exercising and reflecting on the experiences |
|  | [M]editation |
| **Avoidance/Inaction/Rationalization (14%)** | Honestly my moral distress is one reason I do not plan to work in a clinical setting for my career. |
|  | [S]ome of these experiences have deeply changed me and my understanding of the practice of medicine in a deeply troubling way |
|  | My distress hasn't been alleviated; I am just trying to ignore it. |
|  | Haven't had much time to reflect on it. Excused it as being part of the "system." |
|  | Reassure myself that I'll feel better dealing with other populations |
| **Action (6%)** | Try to intervene in the situations where I can |
|  | Tried to speak up except when absolutely inappropriate; airing the concerns did help to relieve the stress, although, as a medical student, you have to be careful about who you choose to air them with |
|  | I attempted to fix the [situation]. I never regretted acting on my instinct, but always regretted when I didn't. |
